# Differences in gene expression profiles for subcutaneous adipose, liver, and skeletal muscle tissues between Meishan and Landrace pigs with different backfat thicknesses

**DOI:** 10.1371/journal.pone.0204135

**Published:** 2018-09-21

**Authors:** Misaki Kojima, Ikuyo Nakajima, Aisaku Arakawa, Satoshi Mikawa, Toshimi Matsumoto, Hirohide Uenishi, Yuki Nakamura, Masaaki Taniguchi

**Affiliations:** 1 Animal Genome Unit, Institute of Livestock and Grassland Science, National Agriculture and Food Research Organization (NARO), Tsukuba, Ibaraki, Japan; 2 Meat Quality Research Unit, Institute of Livestock and Grassland Science, National Agriculture and Food Research Organization (NARO), Tsukuba, Ibaraki, Japan; 3 Animal Bioregulation Unit, Institute of Agrobiological Sciences, National Agriculture and Food Research Organization (NARO), Tsukuba, Ibaraki, Japan; 4 Insect Genome Research Unit, Institute of Agrobiological Sciences, National Agriculture and Food Research Organization (NARO), Tsukuba, Ibaraki, Japan; Universita degli Studi di Bologna, ITALY

## Abstract

Backfat thickness is one of the most important traits of commercially raised pigs. Meishan pigs are renowned for having thicker backfat than Landrace pigs. To examine the genetic factors responsible for the differences, we first produced female crossbred pig lines by mating Landrace (L) × Large White (W) × Duroc (D) females (LWD) with Landrace (L) or Meishan (M) boars (i.e., LWD × L = LWDL for Landrace offspring and LWD × M = LWDM for the Meishan offspring). We confirmed that LWDM pigs indeed had a thicker backfat than LWDL pigs. Next, we performed gene expression microarray analysis in both genetic lines to examine differentially expressed genes (DEGs) in energy metabolism-related tissues, subcutaneous adipose (fat), liver, and *longissimus dorsi* muscle tissues. We analyzed the annotation of DEGs (2-fold cutoff) to functionally categorize them by Gene Ontology and Kyoto Encyclopedia of Genes and Genomes pathways. The number of DEGs in muscle tissues of both lines was much less than that in fat and liver tissues, indicating that DEGs in muscle tissues may not contribute much to differences in backfat thickness. In contrast, several genes related to muscle (in fat tissue) and lipid metabolism (in liver tissue) were more upregulated in LWDM pigs than LWDL pigs, indicating that those DEGs might be responsible for differences in backfat thickness. The different genome-wide gene expression profiles in the fat, liver, and muscle tissues between genetic lines can provide useful information for pig breeders.

## Introduction

Genetic improvements in physiological characteristics related to productivity and quality of meat is an economically important subject matter to livestock producers. Backfat thickness is one of the most important traits of pigs grown for commercial market. Therefore, the study of genetic factors regulating the development of subcutaneous adipose tissues has been intensively investigated [1–3]. Thus far, more than 3,600 quantitative trait loci (QTLs) associated with fat-related phenotypes have been reported in pigs [4]. However, information provided by QTLs has not been sufficient for use in breeding programs because hundreds of genes are contained in QTL regions of DNA [5]. The integration of QTL information and associated physiological information, such as regulation of adipocyte differentiation, would be useful for determining responsive genes in QTL regions [6, 7]. In fact, we have investigated genome-wide gene expression profiles during the adipocyte differentiation of a PSPA cell line, derived from a clonal preadipocyte cell line established from porcine subcutaneous tissues [8], using DNA microarray analysis [9]. The study revealed that several differentially expressed genes identified during adipocyte differentiation co-localized to previously detected fat-related QTL regions, which suggests that these genes are candidates for fat-related QTL regions.

Chinese Meishan pigs are fatter than conventional European breeds, particularly in the amount of subcutaneous adipose tissue they possess [10, 11]. Several QTL studies have been conducted using families of Meishan pigs to identify genetic factors determining their backfat thickness and adiposity degree. Further, the responsible QTLs and/or candidate genes for the traits have been reported by several studies [12–18]. Rather than employing the genetic QTL approach, we focused on detecting differences in the cellularity of adipose tissues between the Meishan and Landrace pigs. We observed that compared with Landrace pigs, Meishan pigs have larger adipocytes in subcutaneous adipose tissues; this may explain why Meishan pigs have thicker backfat tissue [19]. Recently, a genome-wide gene expression analysis has been successfully performed using target tissues of pig breeds that widely vary in objective traits; the analysis revealed that genetic factors responsible for those objective traits are related to differences in gene expression profiles [20–24]. However, no differences in gene expression profiles were observed between Meishan and Landrace pigs for any of the examined tissues, including tissue obtained from subcutaneous adipose.

This study aimed to gain a better understanding of genetic factors responsible for differences in backfat thickness between Meishan and Landrace pigs. Therefore, we investigate differences in gene expression profiles between female crossbred pig lines derived from Landrace and Meishan pigs using DNA microarray analysis with tissues important for energy metabolism [25, 26]. We also discuss the physiological significance caused by differentially expressed genes observed in our present study.

## Materials and methods

### Animals

Crossbred female offspring were produced by mating 2 heads of Landrace (L) × Large White (W) × Duroc (D) females (LWD) with one Landrace (L) or Meishan (M) boar (i.e., LWD × L = LWDL for the Landrace offspring and LWD × M = LWDM for the Meishan offspring). The pigs were maintained at the Institute of Livestock and Grassland Science, National Agriculture and Food Research Organization (NARO). They were fed a diet of commercial grain and provided with water *ad libitum*. The pigs were slaughtered between 10:00 and 11:00 am by electrical stunning, followed by exsanguination. The slaughtered pigs were belonged to one of the following three growth stages: 85-day-old fetuses in late pregnancy (6 each of LWDL and LWDM pigs), 12-day-olds in the suckling stage (5 LWDL and 6 LWDM pigs), and 5-month-olds in the fattening stage (8 LWDL and 7 LWDM pigs). The 85-day-old fetuses were sampled after the 12-day- and 5-month-old pigs. Only female fetuses, which were removed from their mothers on gestational day 85, were collected. Backfat thickness was measured at mid-dorsal area, and the average of those measurements was used to define backfat thickness of each pig. Subcutaneous adipose tissue (fat), liver tissue, and *longissimus dorsi* muscle (muscle) tissue were dissected, immediately frozen in liquid nitrogen, and then stored at −80°C until further use. This study was conducted in strict accordance with the guidelines issued by the Institute of Livestock and Grassland Science, NARO for the care and use of laboratory animals. Extensive efforts were made to minimize suffering in the animals used.

### Measurement of serum biochemical components

Blood samples were collected from individual pigs between 10:00 and 11:00 am. After allowing to clot at room temperature, the serum was separated from each blood sample by centrifuging at 1500 *g* for 15 min at 4°C and then stored at −80°C until use. Total cholesterol (TCHO), triglyceride (TG), and glucose levels in the serum were measured at Oriental Yeast Co., LTD (Tokyo, JAPAN) using a 7020 Automatic Analyzer (Hitachi, Tokyo, Japan).

### Measurement of hepatic TG and TCHO content

Hepatic TG and TCHO content were measured at Skylight Biotech (Akita, Japan) using the Folch method [27] with Cholestest TG and Cholestest CHO kits (Sekisui Medical, Tokyo, Japan), respectively. The values were standardized to 1 g of liver weight.

### Expression analysis using pig oligomer DNA microarray

The pig DNA microarray, AGPOA3 (including 43,221 of 60-base oligonucleotide probes) was prepared by Agilent technologies, Inc., CA, USA. For the most appropriate approach for examining genetic factors for differences in backfat thickness, the AGPOA3 was improved from the AGPOA2 used in a previous study [9] by updating the gene sequence information of pig genome Sscrofa10.2 (http://Aug2014.archive.ensembl.Org/Sus_scrofa/Info/Index).

Each tissue was crushed under liquid nitrogen using the CP-100W CRYO-PRESS (Microtec Co. Ltd., Chiba, Japan). Total RNA was extracted from each tissue using the RNeasy Midi Kit (QIAGEN, Hilden, Germany). Total RNA was measured using the NanoDrop1000 Spectrophotometer (Thermo Fisher Scientific, Wilmington, DE. USA) and its quality was evaluated using Agilent RNA 6000 Nano Kit on an Agilent 2100 Bioanalyzer System (Agilent Technologies, Santa Clara, CA. USA). RNA Integrity Number of all RNA samples were >7.0. Each total RNA (400 ng) was used to synthesize cyanine (Cy) 3-labeled complementary RNA (cRNA) using a two-color, QuickAmp Labeling kit (Agilent Technologies). A mixture of equal amounts of total RNA prepared from the liver of two female LWD pigs was used as the internal control. The resulting cRNA was labeled using Cy5. Labeled cRNA was purified using an RNeasy Mini Kit (QIAGEN). A mixture of equal amounts of Cy3- and Cy5-labeled cRNA (850 ng each) was hybridized [using a Gene Expression Hybridization Kit (Agilent Technologies)] to each array in Agilent’s SureHyb Hybridization Chambers at 65°C for 17 h under constant rotation. Each array was washed with Gene Expression Wash Pack (Agilent Technologies). The microarrays were scanned at 5 μm/pixel resolution using an Agilent DNA Microarray Scanner (G2505B). All kits and products were used in accordance with the manufacturer’s instructions.

### Analysis of microarray data

Raw data were processed using Feature Extraction software Version 9.5 (Agilent Technologies) as previously described [9]. Spot data identified by the software as greater than the background were used for additional analyses. The resulting MIAME compliant microarray data were submitted to the NCBI Gene Expression Omnibus database repository (http://www.ncbi.nlm.nih.gov/geo/) under the accession number **GSE97711**.

Gene expression profiles were analyzed with Subio Basic-Plug-in software (Subio Inc., Kagoshima, Japan). Differentially expressed genes (DEGs) between LWDL and LWDM pigs were defined as a >2.0-fold expression difference with a false discovery ratio (FDR) of < 0.05 by the Benjamini-Hochberg procedure [28], as determined using a student’s *t*-test. Functional annotations of DEGs were investigated with the Gene Ontology Biological Process (GOBP) and with the Kyoto Encyclopedia of Genes and Genomes (KEGG) pathway analyses using the Database for Annotation, Visualization and Integrated Discovery (DAVID) v6.7 [29, 30]. Any statistically significant GOBP terms and KEGG pathways (EASE-score, a modified Fisher’s exact *P*-value < 0.01) we found were selected for further analysis.

## Results

### Features of the pig DNA microarray platform AGPOA3

The AGPOA3 consisted of 43,221 probes corresponding to 16,331 human transcripts associated with human Entrez Gene IDs and/or 16,211 DAVID annotations and 180 probes derived from an unknown gene. Therefore, each annotated gene could be covered with about 2.7 probes on the microarray. The 16,211 genes with DAVID annotations for humans were categorized in 12 sub-clusters of GOBP, six molecular functions, and eight cellular components. This indicated that the AGPOA3 microarray covered many functional genes (S1 Fig).

### Physiological characteristics in LWDL and LWDM pigs

Body weight, backfat thickness, and serum and hepatic levels of biological components of the studied pigs are shown in Table 1. At 12 days of age (suckling stage), LWDL pigs were significantly heavier than the LWDM pigs. However, there were no significant differences in body weight between LWDL and LWDM pigs of either the 85-day-old fetuses or 5-month-old (fattening) pigs. LWDL sucklings had significantly thicker backfat than LWDM sucklings; however, the reverse was true for fattening pigs (i.e., the backfat of LWDM fattening pigs was 1.5-fold thicker than that of LWDL fattening pigs). Serum and hepatic TG levels were significantly higher in LWDM pigs than in LWDL sucklings and fattening pigs. In contrast, serum glucose levels were significantly higher in LWDL than in LWDM sucklings and fattening pigs. Serum and hepatic TCHO levels of LWDM sucklings were significantly 2.5-fold and 1.7-fold higher, respectively, than those of LWDL sucklings. There were no significant differences in serum or hepatic TCHO between crossbred pigs at other ages (i.e. fetus or fattening pigs).

**Table 1:** Physiological characteristics in LWDL and LWDM pigs at the age of 85-day-old of fetus, 12-day-old or 5-month-old.

Age	Breed type ^a^	
Measured parameter	LWDL	LWDM
***Fetus*** (85-day-old)	(n = 6)	(n = 6)
Body weight (g)	492.6 ± 87.2	479.5 ± 53.6
Lipid contents in the liver		
TCHO (mg /g tissue)	2.4 ± 0.1	2.3 ± 0.2
TG (mg / g tissue)	2.0 ± 0.5	1.5 ± 0.4
***Suckling*** (12-day-old)	(n = 5)	(n = 6)
Body weight (kg)	4.4 ± 0.3	3.1 ± 0.6**
Backfat (mm)	2.2 ± 0.3	1.2 ± 0.5**
Serum component		
TCHO (mg / dl)	95.0 ± 26.0	239.5 ± 16.9**
TG (mg / dl)	48.2 ± 14.8	92.3 ± 21.9**
GLU (mg / dl)	150.0 ± 17.4	130.3 ± 10.1*
Lipid contents in the liver		
TCHO (mg / g tissue)	3.0 ± 0.5	5.0 ± 0.4**
TG (mg / g tissue)	2.4 ± 0.8	5.1 ± 1.0**
***Fattening*** (5-month-old)	(n = 8)	(n = 7)
Body weight (kg)	82.5 ± 3.8	82.2 ± 6.6
Backfat (mm)	16.1 ± 2.2	25.6 ± 4.8**
Serum component		
TCHO (mg / dl)	85.1 ± 9.0	95.0 ± 13.2
TG (mg / dl)	33.0 ± 8.3	55.7 ± 22.1**
GLU (mg / dl)	94.0 ± 6.7	81.9 ± 3.4**
Lipid contents in the liver		
TCHO (mg / g tissue)	2.1 ± 0.2	2.0 ± 0.2
TG (mg / g tissue)	2.8 ± 0.2	3.7 ± 0.6**

^a^In the columns of breed type, numbers of pigs used in the group are indicated in brackets. Abbreviations: TCHO, total cholesterol; TG, triglyceride; GLU, glucose. Asterisks indicate significant differences between LWDL and LWDM pigs assessed by Student’s *t*-test

*P < 0.05

**P < 0.01.

### Number of DEGs between LWDL and LWDM pigs

The number of DEGs between LWDL and LWDM is summarized in Table 2. Approximately 40% of the 43,221 probes on the AGPOA3 microarray qualified as expressed probes in each tissue type. Of those 40%, differentially expressed probes between LWDL and LWDM pigs were approximately 0.5%–2% in fat tissue, 1.3%–2.2% in liver tissue. In muscle, 1.1%–1.7% of probes were differentially expressed in fattening pigs (5-month-old) and fetus (85-day-old), but no differentially expressed probes were observed in suckling (age, 12 days). These differentially expressed probes corresponded to the human Entrez Gene ID. The lists of DEGs with the human Entrez Gene ID in fat, liver, and muscle tissues for LWDL and LWDM pigs are provided in S1–S3 Tables. There were no DEGs in muscle tissues of sucklings of both lines, and the fat of 5-month-old pigs of both genetic lines contained lesser DEGs than the other tissues of all stages did. To extract biological significances in DEGs, GOBP and KEGG pathway analyses (*P* < 0.01) were performed for DEGs with human Entrez Gene ID, and Gene Symbols included in the GOBP terms and KEGG pathways are provided in Tables 3–6.

**Table 2:** Summary of differentially expressed gene profiles in the subcutaneous adipose (fat), liver, and *longissimus dorsi* muscle (muscle) tissues between LWDL and LWDM pigs.

Measurement items	Fat		Liver		Muscle	
	LWDM	LWDL	LWDM	LWDL	LWDM	LWDL
**Total number of probes expressed in each tissue**						
	19,942		17,011		19,222	
**Number of over the 2.0-fold higher expressed probes in each breed**						
fetus 85-day-old	na	na	221	213	217	325
12-day-old	342	403	225	219	0	0
5-month-old	107	107	372	381	283	245
**Number of over the 2.0-fold higher expressed genes corresponding to human Entrez Gene ID**						
fetus 85-day-old	na	na	136	135	145	129
12-day-old	224	211	137	145	0	0
5-month-old	73	62	233	215	177	131
**Number of GOBP term (*P* < 0.01) in over the 2-fold higher expressed genes**						
fetus 85-day-old	na	na	2	1	1	2
12-day-old	8	6	3	8	0	0
5-month-old	3	0	4	3	2	0
**Number of KEGG pathway (*P* < 0.01) in over the 2-fold differentially expressed genes**						
fetus 85-day-old	na	na	0	0	0	0
12-day-old	3	1	2	3	0	0
5-month-old	0	0	2	4	1	0

na: not available.

**Table 3:** Annotation profile of the genes with >2.0-fold predominant expression in the subcutaneous adipose tissue (fat) of LWDM and LWDL pigs.

GOBP ID and Term	*P*-Value*^1^	HsGene Symbol
***12-day-old***		
***LWDM***		
GO:0030049 muscle filament sliding	1.13E-08	*MYL2*, *MYL1*, *MYH4*, *ACTA1*, *MYH7*, *TNNT3*, *MYH8*, *TTN*, *TNNI2*
GO:0006936 muscle contraction	4.67E-06	*MYL1*, *MYH4*, *ACTA2*, *ACTA1*, *MYH7*, *CALD1*, *MYH8*, *TMOD2*, *TTN*, *MYLPF*
GO:0006695 cholesterol biosynthetic process	7.74E-05	*INSIG1*, *MVD*, *ACLY*, *HMGCR*, *EBP*, *IDI1*
GO:0060048 cardiac muscle contraction	1.88E-03	*MYL2*, *MYL1*, *MYH7*, *TTN*, *TNNI2*
GO:0003009 skeletal muscle contraction	2.68E-03	*MYH7*, *TNNT3*, *MYH8*, *TNNI2*
GO:0046034 ATP metabolic process	6.14E-03	*MYH4*, *MYH7*, *MYH8*, *HSPA8*
GO:0030239 myofibril assembly	9.81E-03	*MYOZ3*, *TMOD2*, *MYOZ1*
GO:0006941 striated muscle contraction	9.81E-03	*PGAM2*, *MYH7*, *TTN*
***LWDL***		
GO:0019229 regulation of vasoconstriction	1.19E-03	*ADRA2A*, *PER2*, *AGT*, *ADRA1A*
GO:0010863 positive regulation of phospholipase C activity	3.87E-03	*PDGFRA*, *FGF2*, *KIT*
GO:0043552 positive regulation of phosphatidylinositol 3-kinase activity	4.30E-03	*ERBB4*, *PDGFRA*, *FGF2*, *KIT*
GO:0014068 positive regulation of phosphatidylinositol 3-kinase signaling	5.10E-03	*ERBB4*, *AGT*, *PDGFRA*, *KIT*, *PRR5*
GO:0048015 phosphatidylinositol-mediated signaling	5.55E-03	*ERBB4*, *PDGFRA*, *FGF2*, *BTC*, *KIT*, *PRR5*
GO:0014066 regulation of phosphatidylinositol 3-kinase signaling	9.66E-03	*ERBB4*, *PDGFRA*, *FGF2*, *BTC*, *KIT*
***5-month-old***		
***LWDM***		
GO:0060065 uterus development	1.19E-03	*RBP4*, *HOXA10*, *HOXA9*
GO:0009611 response to wounding	1.63E-03	*SLC1A2*, *NRP1*, *SULF2*, *CTGF*
GO:0048706 embryonic skeletal system development	5.46E-03	*RBP4*, *SULF2*, *HOXA9*

*^1^: *P*-value is obtained by Fisher’s exact test.

No GOBP terms were detected in 5 months old LWDL pigs.

**Table 4:** Profile of Kyoto Encyclopedia of Genes and Genomes (KEGG) pathway for 2-fold higher expressed genes in the subcutaneous adipose (fat), liver and *longissimus dorsi* muscle (muscle) tissues of LWDM and LWDL pigs.

Tissue	Ages Breeds	KEGG pathway	*P*-value*^1^	HsGene Symbol
Fat^*2^	***12-day-old***			
	***LWDM***	hsa04530:Tight junction	1.75E-04	*MYL2*, *SHROOM3*, *NRAS*, *MYH4*, *MYH7*, *MYH8*, *ACTG1*, *ACTN4*, *GNAI3*, *MYLPF*
		hsa01100:Metabolic pathways	2.52E-03	*PDHB*, *PTGES*, *AGL*, *GPAM*, *LIPG*, *RIMKLA*, *CMPK1*, *HMGCR*, *NAMPT*, *GLCE*, *NT5E*, *INPP4A*, *PON3*, *ADH1C*, *PGAM2*, *ACLY*, *EBP*, *RDH16*, *MMAB*, *PNPLA3*, *MTMR7*, *NDUFC2-KCTD14*, *AMACR*, *CHDH*, *CYCS*, *CSAD*, *MVD*, *PYGM*, *MCCC2*, *IDI1*, *CYP26B1*
		hsa05416:Viral myocarditis	9.72E-03	*CYCS*, *MYH7*, *ACTG1*, *HLA-A*, *HLA-DRB1*
	***LWDL***	hsa00980:Metabolism of xenobiotics by cytochrome P450	6.86E-03	*ALDH3B1*, *GSTM3*, *CBR3*, *GSTK1*, *CYP3A4*
Liver^*2^	***12-day-old***			
	***LWDM***	hsa03320:PPAR signaling pathway	1.92E-04	*LPL*, *ACOX1*, *CPT2*, *CD36*, *FADS2*, *PCK1*
		hsa01100:Metabolic pathways	1.00E-03	*AADAT*, *CYP3A4*, *ACOX1*, *CYP2J2*, *COQ7*, *PCK1*, *MCCC2*, *G6PC*, *PANK3*, *GANC*, *HMGCS2*, *ENO2*, *CYP4F3*, *UGT8*, *CYP4F2*, *RDH16*, *IDI1*, *ATP6V0A4*, *OAT*, *PON3*, *AOC3*
	***LWDL***	hsa00900:Terpenoid backbone biosynthesis	2.45E-06	*MVD*, *HMGCR*, *FDPS*, *HMGCS1*, *ACAT2*, *IDI1*
		hsa00100:Steroid biosynthesis	4.63E-05	*EBP*, *MSMO1*, *SQLE*, *CYP51A1*, *FDFT1*
		hsa01130:Biosynthesis of antibiotics	3.23E-04	*MSMO1*, *MVD*, *SQLE*, *HMGCR*, *CYP51A1*, *FDPS*, *HMGCS1*, *ACAT2*, *IDI1*, *FDFT1*
	***5-month-old***			
	***LWDM***	hsa01100:Metabolic pathways	1.20E-03	*UQCRC2*, *ACOX1*, *ACADSB*, *CYP2J2*, *ALG8*, *ATP6V1B1*, *CKB*, *MCCC2*, *CSAD*, *DHODH*, *FASN*, *UGT8*, *ACSL4*, *MTMR7*, *B4GALT4*, *AADAT*, *ACSM2A*, *ACMSD*, *AK7*, *PNPLA3*, *CTH*, *PANK3*, *GCK*, *CYP4F3*, *GPT*, *ALOX5*, *CYP4F2*, *RDH16*, *OAT*, *PON3*, *MPST*, *PRODH*
		hsa01212:Fatty acid metabolism	5.29E-03	*ACOX1*, *ACADSB*, *SCD*, *FASN*, *ACSL4*
	***LWDL***	hsa05204:Chemical carcinogenesis	1.97E-04	*CYP3A4*, *GSTA3*, *GSTM3*, *UGT2B17*, *CYP2C18*, *UGT2A2*, *UGT2A1*
		hsa00982:Drug metabolism–cytochrome P450	7.58E-04	*CYP3A4*, *GSTA3*, *GSTM3*, *UGT2B17*, *UGT2A2*, *UGT2A1*
		hsa00980:Metabolism of xenobiotics by cytochrome P450	1.11E-03	*CYP3A4*, *GSTA3*, *GSTM3*, *UGT2B17*, *UGT2A2*, *UGT2A1*
		hsa00830:Retinol metabolism	5.01E-03	*CYP3A4*, *UGT2B17*, *CYP2C18*, *UGT2A2*, *UGT2A1*
Muscle^*2^	***5-month-old***			
	***LWDM***	hsa00500:Starch and sucrose metabolism	3.10E-03	*PGM1*, *HK2*, *AMY2B*, *AMY2A*

*^1^: *P*-value is obtained by Fisher’s Exact test.

^*2^: The categories not described are the categories where KEGG pathways were not detected.

**Table 5:** Annotation profile of the genes with >2.0-fold predominant expression in the liver tissue of LWDM and LWDL pigs.

GOBP ID and Term	*P*-Value*^1^	HsGene Symbol
***Fetus 85-day-old***		
***LWDM***		
GO:0006690 icosanoid metabolic process	1.38E-03	*CYP2J2*, *CYP4F3*, *CYP4F2*
GO:0019373 epoxygenase P450 pathway	7.19E-03	*CYP2J2*, *CYP2C18*, *CYP4F2*
***LWDL***		
GO:0008584 male gonad development	3.97E-03	*LRRC6*, *TNFSF10*, *BCL2*, *ESR1*, *NR0B1*
***12-day-old***		
***LWDM***		
GO:0006690 icosanoid metabolic process	1.36E-03	*CYP2J2*, *CYP4F3*, *CYP4F2*
GO:0055085 transmembrane transport	1.77E-03	*ABCC9*, *SLC25A25*, *SLC16A6*, *SLC25A23*, *MFSD2A*, *SLC47A1*, *SLC43A1*, *SLC47A2*
GO:0006629 lipid metabolic process	5.21E-03	*CYP3A4*, *LPL*, *ACOX1*, *CD36*, *FADS2*, *RDH16*
***LWDL***		
GO:0006695 cholesterol biosynthetic process	1.69E-13	*EBP*,* MSMO1*,* MVD*,* SQLE*,* HMGCR*, *CYP51A1*,* INSIG1*,* FDPS*,* HMGCS1*, *IDI1*,* FDFT1*
GO:0008299 isoprenoid biosynthetic process	4.33E-08	*MVD*,* HMGCR*,* FDPS*,* HMGCS1*,* IDI1*,* FDFT1*
GO:0008203 cholesterol metabolic process	1.63E-04	*APOL2*,* EBP*,* LDLR*,* SQLE*,* INSIG1*,* SREBF2*
GO:0006629 lipid metabolic process	1.91E-04	*APOL2*,* FAR2*,* LDLR*,* HMGCS1*,* ACSL4*,* AACS*,* ACAT2*,* SREBF2*
GO:0070098 chemokine-mediated signaling pathway	2.05E-03	*CCL2*,* CCR5*,* CCL8*,* CCL19*,* XCR1*
GO:0002250 adaptive immune response	5.35E-03	*PIK3CG*,* CD244*,* SH2D1A*,* THEMIS*,* TAP1*,* EOMES*
GO:0002407 dendritic cell chemotaxis	7.17E-03	*PIK3CG*,* CCR5*,* CCL19*
GO:0006954 inflammatory response	8.18E-03	*PIK3CG*,* CCL2*,* CCR5*,* CCL8*,* CCL19*,* XCR1*,* PLA2G2D*,* IGFBP4*,* CD180*
***5-month-old***		
***LWDM***		
GO:0045926 negative regulation of growth	1.26E-03	*MT1L*, *MT2A*, *MT1E*, *MT1F*
GO:0071294 cellular response to zinc ion	1.26E-03	*MT1L*, *MT2A*, *MT1E*, *MT1F*
GO:0006690 icosanoid metabolic process	3.52E-03	*CYP2J2*, *CYP4F3*, *CYP4F2*
GO:0055114 oxidation-reduction process	3.54E-03	*STEAP3*, *CYP2J2*, *SCD*, *CYB5B*, *SESN3*, *FMO3*, *MIOX*, *DHODH*, *FASN*, *CHM*, *MPO*, *CYP4F3*, *ALOX5*, *CYP4F2*, *RDH16*, *PRODH*
***LWDL***		
GO:0006954 inflammatory response	1.56E-04	*LY75*, *CIITA*, *C5*, *CRP*, *CCL19*, *CCL8*, *IL34*, *CD180*, *CCL26*, *SCN9A*, *CLEC7A*, *PTX3*, *PLA2G2D*, *AOC3*
GO:0010634 positive regulation of epithelial cell migration	4.67E-03	*JUN*, *VIL1*, *ITGA2*, *CLASP1*
GO:0007155 cell adhesion	8.10E-03	*AMBN*, *CD34*, *MPDZ*, *COL6A5*, *CNTNAP2*, *ITGA2*, *COL8A1*, *PCDH17*, *ITGBL1*, *EPHA3*, *CDH6*, *AOC3*

*^1^: *P*-value is obtained by Fisher’s exact test.

**Table 6:** Annotation profile of the genes with >2.0-fold predominant expression in the skeletal muscle (muscle) tissue of LWDM and LWDL pigs.

GOBP ID and Term	*P*-Value*^1^	HsGene Symbol
***Fetus 85-day-old***		
***LWDM***		
GO:0030199 collagen fibril organization	2.33E-03	*ACAN*,* LOX*,* ADAMTS3*,* GREM1*
***LWDL***		
GO:0042523 positive regulation of tyrosine phosphorylation of Stat5 protein	2.14E-04	*ERBB4*,* PECAM1*,* KIT*,* GHR*
GO:0008283 cell proliferation	1.12E-03	*EPS15*,* AMBN*,* EPS8*,* CDC14A*,* ERBB4*,* NASP*,* BCL2*,* TGFBI*,* IRF2*,* TCF7L2*
***5-month-old***		
***LWDM***		
GO:0002931 response to ischemia	3.31E-03	*RNLS*, *PANX2*, *UCHL1*, *HK2*
GO:0060333 interferon-gamma-mediated signaling pathway	4.01E-03	*HLA-DRB1*, *HLA-A*, *TRIM26*, *OAS1*, *HLA-DQA1*

*^1^: *P*-value is obtained by Fisher’s exact test.

No GOBP terms were detected in 12 days old LWDM and LWDL and 5 months old LWDL pigs.

### Characteristics of DEGs in fat

More GOBP terms were obtained in the fat of LWDM and LWDL sucklings than of fattening pigs (Table 2). We observed several muscle-related GOBP terms [including “muscle filament sliding” (GO:0030049), “muscle contraction” (GO:0006936), and “tight junction” KEGG pathway (hsa04530)] in LWDM sucklings. Genes coding for actin isoforms, such as actin gamma 1 (ACTG1), and myosin isoforms, such as myosin light chain 2 (MYL2), were identified in those GOBP terms and/or the KEGG pathway (Tables 3 and 4). Such muscle-related GOBP terms and KEGG pathways were not detected in the fat of fattening LWDM pigs or all stages in LWDL pigs.

We further observed “cholesterol biosynthetic process” (GO:0006695) in the fat of LWDM sucklings. A part of the genes including *3-hydroxy-3-methylglutaryl-CoA reductase* (*HMGCR*) in the GOBP term were observed in the “metabolic pathway” (hsa01100) KEGG pathway (Table 4).

Several signaling-related GOBP terms [including“positive regulation of phospholipase C activity” (GO:0010863) and “positive regulation of phosphatidylinositol 3-kinase signaling” (GO:0014086)] were observed in LWDL sucklings but not in LWDM sucklings. The genes, such as *angiotensinogen* (*AGT*), *fibroblast growth factor 2* (*FGF2*), and *platelet-derived growth factor receptor alpha* (*PDGFRA*) were detected in those GOBP terms observed in LWDL sucklings (Table 3).

### Characteristics in DEGs in the liver

We observed several GOBP terms and KEGG pathways related to metabolic processes in the liver of all examined pigs (Tables 4 and 5). In all age of the examined LWDM pigs, we observed the “icosanoid metabolic process” (GO:0006690) expressed by the *Cytochrome P450 (CYP) 2J2*, *CYP4F2* and *CYP4F3* genes (Table 5). Furthermore, “fatty acid metabolism pathway (hsa01212) was identified in fattening (5-month-old) LWDM pigs (Table 4). In this pathway, both fatty acid biosynthesis-related genes [*fatty acid synthase* (*FASN*), *acyl-CoA synthetase long-chain family member 4* (*ACSL4*) and *stearoyl-CoA desaturase* (*SCD*)] and the fatty acid β-oxidation-related genes [*acyl-CoA oxidase 1* (*ACOX1*), *acyl-CoA dehydrogenase* and *short/branched chain* (*ACADSB*)] were identified as upregulated genes in fattening LWDM pigs. Likewise, fatty acid β-oxidation related genes, *carnitine palmitoyltransferase 2* (*CPT2*) and *ACOX* [found in “PPAR signaling pathway” (hsa03320) in Table 4], were also upregulated in sucklings (12-day-old) LWDM pigs.

The “cholesterol biosynthetic process” (GO0006695) and “terpenoid backbone biosynthesis” (hsa00900) were characteristic of LWDL sucklings, but not of fetuses and fattening pigs (Tables 4 and 5). A series of cholesterol biosynthesis-related genes, including *mevalonate (diphospho) decarboxylase* (*MVD*), *3-hydroxy-3-methylglutaryl-CoA synthase 1* (*HMGCS1*), *HMGCR*, *farnesyl-diphosphate farnesyltransferase 1* (*FDFT1*), *farnesyl-diphosphate synthase* (*FDPS*), *squalene epoxidase* (*SQLE*), and *CYP51A1*, were extracted from LWDL sucklings. In LWDL fattening pigs, drug metabolism-related terms such as “Chemical carcinogenesis” (hsa05204) and “Drug metabolism–cytochrome P450” (hsa00982) KEGG pathways were characteristic, and a part of the genes (*CYP3A4*, *UGT2B17* and *CYP2C18*) extracted from these KEGG pathways were also extracted to “retinol metabolism” (hsa00830) KEGG pathway (Table 4).

### Characteristics in DEGs in muscle

Fewer DEGs were found in muscle tissue than in fat or liver tissues in all the examined pigs (Table 2). Particularly, no DEGs were observed in any sucklings. In LWDL fetuses, *growth hormone receptor* (*GHR*), which included in the “positive regulation of tyrosine phosphorylation of Stat5 protein” (GO:0042523), was upregulated. In fattening LWDM pigs, a part of glucose metabolism-related gene was extracted to “starch and sucrose metabolism” (hsa00500) (Tables 4 and 6).

## Discussion

Chinese Meishan pigs are widely recognized as being fatter and having thicker backfat than European Landrace pigs [10, 11]. In our investigation of possible genetic factors responsible for these differences between the two genetic lines, we examined differences in gene expression profiles between crossbred Landrace (L) × Large White (W) × Duroc (D) females (LWD) with Landrace (L) or Meishan (M) pigs (LWDL and LWDM, respectively). Fattening (5-month-old) LWDM pigs had thicker backfat than LWDL fattening pigs, confirming that LWDM pigs inherited the trait for backfat thickness from their Meishan and Landrace parents. Because suckling (12-day-old) LWDM pigs had a thinner backfat and lower body weight than LWDL sucklings, we infer that growth is delayed in LWDM pigs compared with LWDL pigs. In contrast to backfat thickness, serum and hepatic TG levels (a marker of adiposity) were higher in both suckling and fattening LWDM pigs than in LWDL pigs of the same age, thus indicating that the trait selecting for adiposity in Meishan and Landrace pigs is inherited by LWDM and LWDL pigs, respectively.

Because backfat thickness was clearly thicker in fattening LWDM pigs than in fattening LWDL pigs, we presume that adipocyte size would also be larger in LWDM pigs owing to the larger amount of lipid accumulating their thicker backfat [19]. Surprisingly, lipid synthesis/metabolism-related GOBP terms and KEGG pathways were not representatively detected in the fat of any LWDM pigs (all ages) examined, even though they are thought to play key roles in fat formation. Instead, we detected several muscle-related terms such as “muscle filament sliding” (GO:0030049), “muscle contraction” (GO:0006936), and “tight junction” (hsa04530), in the fat of LWDM sucklings. However, those terms were not detected in fattening LWDM pigs or in the fat of LWDL pigs (any age). Because G-actin dynamics trigger adipocyte differentiation [31], the genes coding for actin and myosin isoforms, such as *ACTG1* and *MYL2*, respectively, are components of the muscle-related terms detected in LWDM sucklings, and would, if upregulated in the fat of sucklings, be expected to play important roles for the development of adiposity and backfat thickness. Our findings corroborate with those of Vincent et al. [22], who reported that cytoskeleton-related genes are upregulated in the fat of Basque pigs, which have thicker backfat than Large White pigs. In the fat of LWDL sucklings, the *AGT*, *FGF2*, and *PDGFRA* genes were upregulated. Because these genes are reported to affect adipocyte differentiation [32–35], these findings suggest that these upregulated genes, by affecting adipocyte differentiation, might be involved in promoting thinner backfat in LWDL pigs than in LWDM pigs. Recently, the *malic acid enzyme (ME1)* gene has been reported to be a candidate gene for backfat thickness [24], however, we did not detect the *ME1* gene as DEGs in fat tissues.

In the liver of all examined LWDM pigs (all ages), the *CYP2J2*, *CYP4F2*, and *CYP4F3* genes extracted to “icosanoid metabolic process” (GO:0006690) were upregulated. In the liver of fattening LWDL pigs, the *CYP2C18*, *UGT17B1*, and *CYP3A4* genes extracted to “retinol metabolism” (hsa00830) were upregulated. This observation is consistent with our previous results that those hepatic mRNA levels were higher in Landrace fattening pigs than in Meishan fattening pigs [36, 37]. Furthermore, the gene coding for retinol dehydrogenase 16 (all-trans) (RDH16), an enzyme for retinol metabolism, was upregulated in the liver and fat of suckling and/or fattening LWDM pigs (Table 4). Because retinoic acid, arachidonic acid, and/or their metabolites are known to be agonists for retinoic acid receptors, retinoid X receptors, and/or peroxisome proliferator-activated receptors [38–40], these genes would be partially responsible for differences found in adiposity between LWDL and LWDM pigs (by either producing or eliminating these metabolites).

In the liver of fattening LWDM pigs, fatty acid biosynthesis-related genes [e.g., *FASN*, *SCD*, and *ACSL4* included in “fatty acid metabolism” (hsa01212)] were upregulated. Upregulation of these genes would have been derived from the upregulation of the *sterol regulatory element binding transcription factor 1* (*SREBF1*; S2 Table), which is known to be a key transcriptional factor of lipogenic genes [41]. This observation is supported by our previous study [42], which reported that upregulation of hepatic *SREBF1* mRNA levels occurs in Meishan-derived pigs associated with thick backfat. The *ACSL4* gene has been reported to be a candidate gene for the backfat thickness, because polymorphisms of *ACSL4* are significantly associated with backfat thickness and oleic fatty acid content [18, 43]. The *aminoadipate aminotransferase (AADAT)* gene included in “Metabolic pathway” (hsa01110), involved in producing acetyl-CoA (a precursor for the biosynthesis of fatty acids) from D-lysine [44], was 5~17-fold more highly expressed in all age categories of LWDM pigs (S2 Table). Thus, upregulation of these genes in the liver of LWDM pigs would be expected to promote TG biosynthesis and higher TG contents in both the liver and serum *via* promotion of fatty acid synthesis, which in turn would be expected to promote thicker backfat in LWDM pigs.

Interestingly, the genes involved in the fatty acid β-oxidation process (*ACOX1*, *ACADSB and CPT2*) were also upregulated in the liver of LWDM suckling and fattening pigs. Furthermore, in the liver of LWDM sucklings, the *3-hydroxy-3-methylglutaryl-CoA synthase 2 mitochondrial* (*HMGCS2*), *glucose-6-phosphatase*, *catalytic subunit (G6PC)* and *phosphoenolpyruvate carboxykinase 1* (*PCK1*) genes, coding for rate limiting enzymes for ketone body biosynthesis pathway (*HMGCS2*) and gluconeogenesis (*G6PC* and *PCK1*), were upregulated (Table 4). In LWDM fattening pigs, the *glucokinase* (*GCK*) gene, coding for a rate limiting enzyme for glycolytic processes, was upregulated in the liver (Table 4), and a part of genes related to “starch and sucrose metabolism” (hsa00500) was upregulated in the muscle. From these findings, we suggest that energy utilization in the liver and muscle differs between LWDM and LWDL pigs, especially in sucklings.

A series of cholesterol biosynthesis-related genes were upregulated in the liver of LWDL sucking pigs, and these findings would be derived by upregulation of the gene coding “*sterol regulatory element binding transcription factor 2*” (*SREBF2*) (Table 5) that is the main transcriptional regulator of cholesterogenic genes [42]. The upregulation of these cholesterogenic genes might be resulted from a negative feedback, because the serum and hepatic cholesterol levels were significantly lower in LWDL sucklings than in LWDM sucklings. Contrarily, cholesterogenic genes, including *HMGCR* that encodes the rate-limiting enzyme of the cholesterol biosynthesis pathway, were upregulated in the fat of LWDM sucklings. Because cholesterol is a component of the lipid membrane of cells and is required for cell proliferation, we deduce that more adipocyte proliferation is promoted in the fat of LWDM sucklings than of LWDL pigs, whereas more promotion of growth is occurred in LWDL sucklings than in LWDM sucklings. Based on our results, physiological differences (by at least 12 days of age) may be responsible for differences in backfat thickness and adiposity between LWDM and LWDL pigs.

The DEGs in muscle tissues were much less evident between LWDM and LWDL pigs than in fat and liver tissues, particularly because no DEGs were observed in sucklings. This observation suggests that genes regulating muscle tissue might contribute little to backfat thickness. However, we observed relatively many DEGs in LWDL fetuses, and *GHR* was observed to be upregulated in LWDL fetuses. These results indicate there exist differences in muscle growth in the late pregnancy among breeds, which is supported by studies reporting that western commercialized breeds tend to grow more quickly than Meishan pigs [10, 11].

## Conclusions

We demonstrated differences in genome-wide expression profiles in three tissue types (fat, liver, and muscle) between LWDM and LWDL pigs for three different life stages (fetus, suckling, and fattening). This allowed us to identify genetic differences in backfat thickness and adiposity between Meishan and Landrace breeds. The muscle-related genes in fat tissue and in lipid metabolism-related genes in liver tissue were upregulated in LWDM pigs, suggesting that those genes could be heritable candidates responsible for differences in backfat thickness and adiposity. Furthermore, we suggest that large physiological and gene expression differences in the fat and liver between LWDM and LWDL sucklings might help determine the differences in backfat thicknesses between different lines. The genetic information provided in this study will be helpful for developing DNA markers for breeding programs, although it still remains unclear how each DEG is involved in the regulation of those traits (by tissue and age) between genetically different pig lines.

## Supporting information

S1 Fig Gene ontology annotations of the genes on the AGPOA3 microarray platform. Gene ontology annotation categories of biological process, molecular function, and cellular component were analyzed in 16,211 genes with DAVID annotations derived from 43,221 probes on the AGPOA3 microarray platform. The number in horizontal axis shows the number of the gene annotations.
(DOCX)

S1 Table The >2-fold differentially expressed genes in the subcutaneous adipose tissue between LWDL and LWDM pigs suckling (12-days-old) and fattening (5-months-old). (XLSX)

S2 Table The >2-fold differentially expressed genes in the liver tissue between LWDL and LWDM pigs for 85-day-old fetus, suckling (12-days-old) and fattening (5-months-old). (XLSX)

S3 Table The >2-fold differentially expressed genes in the *longissimus dorsi* muscle tissue between LWDL and LWDM pigs for 85-day-old fetus, suckling (12-day-old) and fattening (5-months-old). (XLSX)
