# Supplementary figures and images for: Differences in gene expression profiles for subcutaneous adipose, liver, and skeletal muscle tissues between Meishan and Landrace pigs with different backfat thicknesses
Source: PLoS One. 2018 Sep 21;13(9):e0204135. doi: 10.1371/journal.pone.0204135 (PMC6150482; doi:10.1371/journal.pone.0204135)

**Supplementary Fig. 1 (Kojima, *et al.*)**


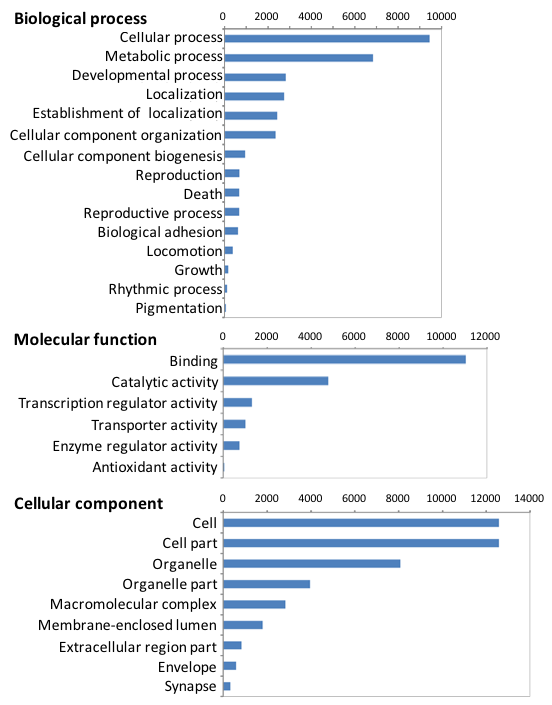

Supplement: S1 Fig — Gene ontology annotation categories of biological process, molecular function, and cellular component were analyzed in 16,211 genes with DAVID annotations derived from 43,221 probes on the AGPOA3 microarray platform. The number in horizontal axis shows the number of the gene annotations. (DOCX) [file pone.0204135.s001.docx]
